# Evaluating the microbial aerosol generated by dental instruments: addressing new challenges for oral healthcare in the hospital infection

**DOI:** 10.1186/s12903-023-03109-5

**Published:** 2023-06-21

**Authors:** Xin Yang, Ruolan Liu, Jiakang Zhu, Tian Luo, Yu Zhan, Chunyuan Li, Yuqing Li, Haiyang Yu

**Affiliations:** 1 State Key Laboratory of Oral Diseases, National Clinical Research Center for Oral Disease, West China Hospital of Stomatology, Sichuan University, Chengdu, 610041 China; 2 Department of Prosthodontics, West China Hospital of Stomatology, Sichuan University, No. 14, 3Rd Section of Ren Min Nan Rd, Chengdu, 610041 Sichuan Province China; 3 Department of Environmental Science and Engineering, Sichuan University, Chengdu, 610065 Sichuan China

**Keywords:** Aerosol, Particles and droplets, Size, Transmission, Threshold limit values, Dental restoration repair, Dental offices

## Abstract

**Background:**

Using a rotary instrument or ultrasonic instrument for tooth preparation is a basic operation in the dental clinic that can produce a significant number of droplets and aerosols. The dental droplet and aerosol can lead to the transfer of harmful germs. The goal of this study was to analyze the properties of microbiological aerosol created by droplets and aerosol generated by three common tooth-preparation instruments.

**Methods:**

*Streptococcus mutans* UA159 was used as the biological tracer to visualize the droplets and aerosols. The passive sampling method was used to map the three-dimensional spatial distribution and the six-stage Andersen microbial sampler (AMS) was used as the active sampling method to catch aerosol particles at a specific time.

**Results:**

The aerosol concentration is related to instruments, three-dimensional spatial distribution, and dissipation time. Most aerosols were generated by air turbines. More microorganisms are concentrated at the 1.5 m plane. The majority of the post dental procedure contamination was detected within the 0–10-min period and it decreased rapidly within 30 min.

**Conclusion:**

This study is conducive to the proposal and improvement of relevant infection control measures in dental procedures and provides a basis for the assessment of measures, reducing the risk of nosocomial infection.

## Introduction

To this day the dental clinic is still an important place for the prevention and control of hospital acquired infection (HAI) because the dental environment has a high risk of infection transmission [1, 2]. There are many dental devices used in dental treatment such as high-speed handpieces, ultrasonic instruments, and three-in-one air/water syringe. When these power instruments work in the patient’s oral cavity, a large number of aerosols and droplets mixed with the patient’s saliva or even blood will be generated during a dental procedure, but the use of these devices is unavoidable in dental operation [3–6]. Besides, the particles of droplets and aerosols produced in dental treatment are so small that they can stay in the air for an extended period before settling on environmental surfaces or entering the respiratory tract [7–10]. Consequently, apart from contact transmission and droplet spread, indirect transmission, and aerosol spread are also easier to happen in the dental clinic environment [11, 12].

As the dental clinic’s most frequently used dental devices, high-speed air turbine handpieces can generate droplets and aerosols with various microorganisms that contribute to airborne microbial contamination [5, 13]. Several studies have shown that the bacterial and aerosol content in the air around the patient’s oral cavity during dental treatment is higher than that without operation [14–17]. Moreover, using a high-speed air turbine handpiece for tooth preparation may lead to more serious droplet and aerosol pollution than ultrasonic instruments [18]. However, some studies showed that the sprays generated by ultrasonic devices mainly settle on the dominant arm of the operator, eyewear, and chest of the patient and a little on the non‐dominant arm and chest of the operator and assistant [19, 20].

Andrei and others have indicated that the range of microbial aerosol in a closed dental clinic could almost spread the whole space [21]. In addition, some further studies have revealed the relationship between the distribution of droplets and aerosol and the distance from the oral cavity. Airborne microbial contamination would decrease with the increasing distance from the center of the pollution source [21–25]. However, these studies are limited in that they only measured microbial aerosol at several specific locations. Nowadays, there are few studies on microbial aerosol in three-dimensional space and even few on the dissipation efficiency of microbial aerosol. The evidence about the three-dimensional distribution and the persistence of airborne microbial contamination will be a focus of prevention and control of infection in oral health care, especially in the face of public health events.

The present study aimed to measure the three-dimensional spatial distribution of microbial aerosol generated by dental common instruments and analyze its dissipation efficiency in the dental clinic. This study aims to guide infection control in oral health care during the epidemic period of infectious diseases and improve the management scheme for the prevention and control of nosocomial infection in daily dental clinical work.

## Methods

Two experimental designs using simulated tooth preparation procedures were conducted in this study: the three-dimensional spatial distribution experiment of microbial aerosol was conducted in a standard single-chair dental treatment room to investigate airborne contamination caused by droplet and aerosol in this setting and the dissipation time experiment was conducted to investigate the persistence of the microbial aerosol. The same single clinical procedure was conducted in both experiments.

### Bacterial preparation

*Streptococcus mutans* was selected as the bacterial tracer to simulate the diffusion of any airborne infective agent which was obtained from the State Key Laboratory of Oral Diseases. *S. mutans* was grown in Brain Heart Infusion (BHI) broth overnight at 37 °C in a 5%-supplemented carbon dioxide environment. The cells were harvested via centrifugation (4,000 g, 15 min), washed twice with sterile phosphate-buffered saline (PBS), and resuspended and diluted in the same buffer. The bacterial suspension was subjected to vortex (Vortex Genius 3; IKA, Germany; 30 s) to disperse bacterial chains and adjusted to 10^8^ CFU/mL standard for experimental use.

### Establishment of experimental model

**Fig. 1:**
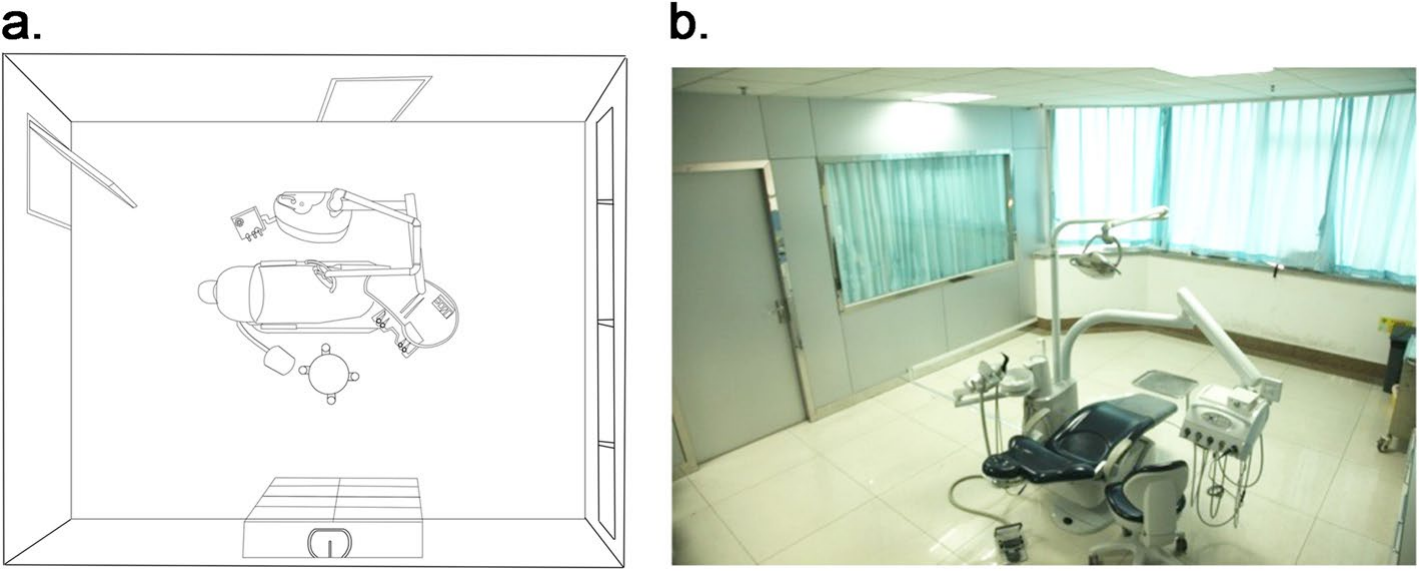
Dental unit model. **a** Sketch map of the single-chair dental treatment room with the coordinate system established. **b** Physical map of the single-chair dental treatment room

Experiments were conducted in a standard single-chair dental treatment room which meets the requirements of secondary biosafety laboratory located in the West China Hospital of Stomatology, Sichuan University, Chengdu, China. This is a 394 cm by 510 cm by 240 cm (length, width, and height, respectively) operative environment equipped with a dental unit (ESTETICATM E80 Vision; KaVo, Germany), a dental chair, and a 4-door cabinet located behind the dental chair (Fig. 1). The air-conditioning system for the operatory was isolated using sealing the inlet. The mechanical ventilation system was off and the door and windows were closed during the experiment [26]. And the dental unit is exposed to ultra-violet and tested by control agar plates to make sure that there are no *S. mutans* before the experiment. The temperature remained constant at 24–26 °C and the humidity at 60%—70%.

**Fig. 2:**
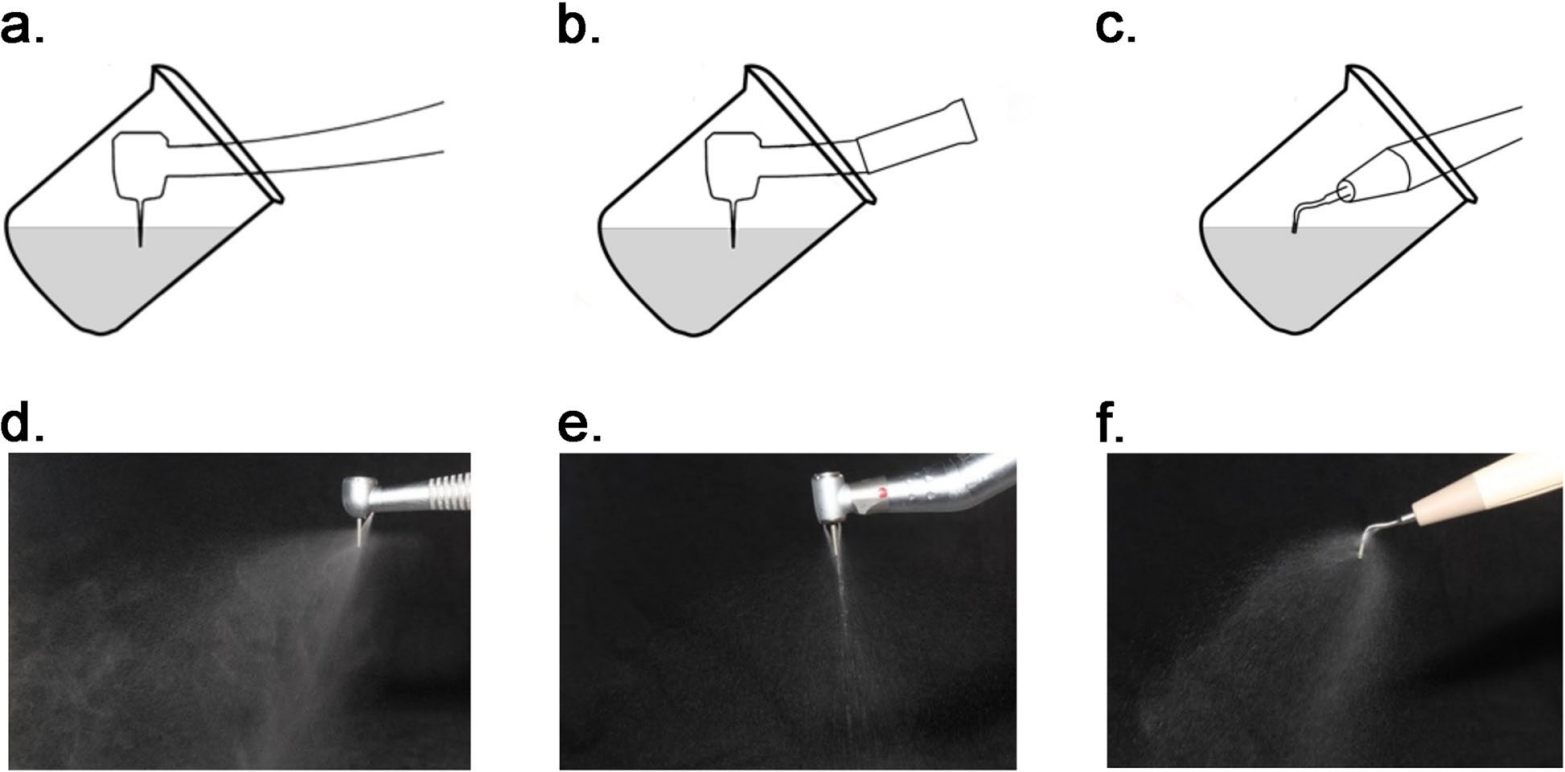
Schemes representing the simulated tooth preparation with half-submerged in the oral environment and operation. **a**, **b** a high-speed air turbine. **c**, **d** a high-speed electric turbine. **e**, **f** an ultrasonic instrument

A dummy head was mounted in a standard working position and was adjusted to make the height of the headrest equal to the operator’s arms with the seat back 45° to the ground. The position of the dummy head’s mouth was 75 cm above the floor. The jaws inside the dummy head oral were taken out and a 50 ml beaker containing 15 ml *S. mutans* suspension was fixed inside. The operator site is similar to the anterior teeth of the patient when the front part of the operator is immersed in the suspension. A high-speed air turbine handpiece (S609C; KaVo, Germany) was used and connected to the dental unit. The rotating speed of the turbine was measured to be 360,000 r/min. To simulate the clinical procedure of tooth preparation, the handpiece was equipped with a cylindrical bur (TR-12; MANI, Japan) which was 10 mm in working length, and the bur was immersed in the bacterial suspension by approximately 5 mm, while the head of the turbine was about 5 mm away from the surface during the experiment [27]. And a high-speed electric handpiece (Restorative classic CA 1:5, Bien-Air, Swiss) was used with motor control (Optima, Bien-Air, Swiss). The rotating speed is 200,000 r/min and the bur is also a cylindrical bur (TR-12; MANI, Japan). An ultrasonic instrument (P5XS Newtron, Satelec, France) with PerfectMarginShoulder(PMS®) tip 1 was 5 mm in working length, and the tip was immersed in the bacterial suspension by approximately 2.5 mm (Fig. 2). The working power was blue light which means high power and amplitude as recommended. The coolant flow rate is 15 mL per minute and excessive liquid will be removed by an aspiration cannula. Only 1 operator and 2 assistants wearing the same biohazard-protective full suits(4565L; 3 M™, USA), N95 masks without valves(9132; 3 M™, USA), goggles(1621AF; 3 M™, USA), face shields, shoe covers, and gloves conducted the procedures.

### Three-dimensional spatial distribution experiments of microbial aerosol

**Fig. 3:**
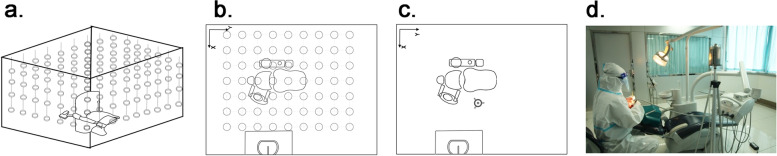
Experimental model. **a** Sketch of the salivarius-bacitracin agar plates in room. **b** Vertical view of sample sites of salivarius-bacitracin agar plates at a height plane. **c** Vertical view of sample site of AMS in the single-chair dental treatment room. **d** Physical map of the single-chair dental treatment room with AMS

The presence of a biological tracer in 252 sites in the operatory was measured for this procedure. According to the length, width, and height of the operatory, 50 cm was set as the unit length, and sampling sites were determined every 50 cm with 4 sampling planes which were set at 0.5 m、1.0 m、1.5 m、2.0 m, 252 sampling sites in totally determined in the whole operatory and the distance between two adjacent sampling sites is 50 cm. Before the beginning of each procedure, 252 mitis salivarius-bacitracin agar plates were suspended by string at the sampling sites and facing upwards while the lids remained closed (Fig. 3a, b). The operator took his position and then 2 assistances opened the lids of every plate. After that, the operator performed a 15-min simulated tooth preparation according to the experimental model. The operator remains in his position and keeps still after the procedure. The plates were closed 15 min after the end of the procedure to allow aerosols to settle and immediately transferred to the microbiological laboratory. The plates were incubated at 37 °C for 48 h in a 5%-supplemented carbon dioxide environment. At the end of the incubation, the colonies were counted and the results were expressed as CFU. Between procedures, the environment was disinfected by spraying 0.5% sodium hypochlorite solution and irradiating ultraviolet rays for 2 h. The same procedure was repeated once using sterile distilled water instead of bacterial suspension as a blank control. This procedure was repeated 20 times; 20 tests and 20 controls.

### Dissipation time experiments of microbial aerosol

In the three-dimensional spatial distribution experiment, the area with the highest tracer level was approximately in the middle of the dental unit, 1.5 m above the ground, and 0.8 m away from the operation site, which was determined to be the sampling site of the experiments (Fig. 3a, b) [13]. During each sampling day, air samples were collected using a six-stage Andersen microbial sampler (AMS; 20–600, Thermo, USA), containing 6 mitis salivarius-bacitracin agar plates sampling sites. The operator performed simulated tooth preparation for 15 min. After that, air samples were taken continuously within 2 h and the mitis salivarius-bacitracin agar plates inside were replaced every 10 min. The sample was collected for 10 min before the baseline level was determined. AMS was operated at 28.3 L/min and the sampling time was 10 min. The experiments were carried out under three experimental conditions after using air turbines, electric turbines, or ultrasonic devices. Therefore, this study allows us to freely compare airborne microbial contamination’s dissipation efficiency after different operations. All plates were incubated at 37 °C for 48 h in a 5%-supplemented carbon dioxide environment. The colonies on levels 1–6 of AMS were counted. Then particle size is analyzed as described previously [28] (Table 1). The positive hole method was used to correct the numbers and the airborne microbial contamination at different times was calculated with the formula (1)

1 $${C}_{i}=\frac{{N}_{i }\times 1000}{t\times Q},$$

where ${C}_{i}$ was airborne microbial contamination, CFU m^−3^; ${N}_{i}$ was the sum of 6 grades effective colonies, CFU; $t$ was the sampling time, min; $Q$ was the flow rate during sampling, L / min [29].

**Table 1:** The particle size ranges for each stage of the Andersen sampler

Stage	Range of Particle Size (Microns)
1	7.0 and above
2	4.7–7.0
3	3.3–4.7
4	2.1–3.3
5	1.1–2.1
6	0.65–1.1

### Statistical analysis

For the three-dimensional spatial distribution experiment, SPSS version 24 (IBM Corp., NY, USA) was used to analyze microbiology data belonging to the tracer presence in the dental treatment room. A Shapiro–Wilk test was applied to check the normality of the data distribution and the Bartlett test was used to check the homogeneity of variances preliminarily. The mean level of each site was used to analyze the three-dimensional spatial distribution of the dentist office. The data were log-transformed to approach a normal distribution. The tracer levels in the planes of different heights were compared through one-way analysis of variance (ANOVA) and Dunnett’s T3 test, at a significance level of 0.05. 3-dimensional graphic models representing the spatial distribution and the biological tracer levels were created using The R Programming Language. 2-dimensional graphic models demonstrating the spatial distribution of biological tracer level in each height plane were created using GraphPad prism 9.

For dissipation time experiments, two-way repeated-measures ANOVA was performed and a posterior comparison among sampling was done using the Bonferroni test. Results were considered significant with *p*-values < 0.05. All statistical tests were two-tailed at a significance level of 0.05. The statistical analyses were also conducted using ORIGIN2021.

## Results

### Three-dimensional spatial distribution of microbial aerosol in the dental treatment room

**Fig. 4:**
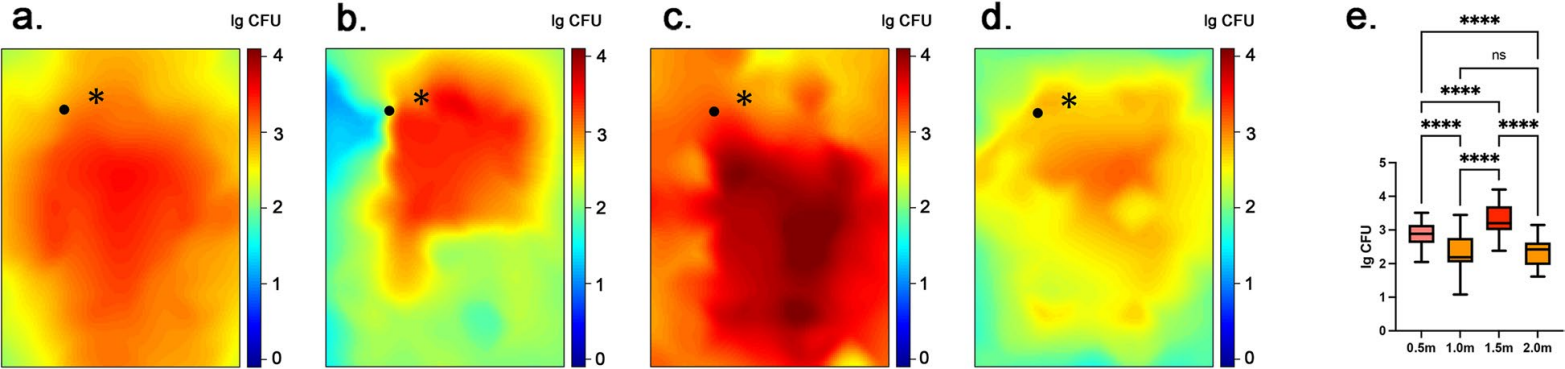
2-dimensional graphic model demonstrating the spatial distribution of biological tracer level in each height plane with a high-speed air turbine: **a** 0.5 m. **b** 1.0 m. **c** 1.5 m. **d** 2.0 m. **e** the biological tracer level in each height plane with a high-speed air turbine. * in each graph represents the location of the infection source, that is, the mouth of the dummy head. • in each graph represents the location of the operator

**Fig. 5:**
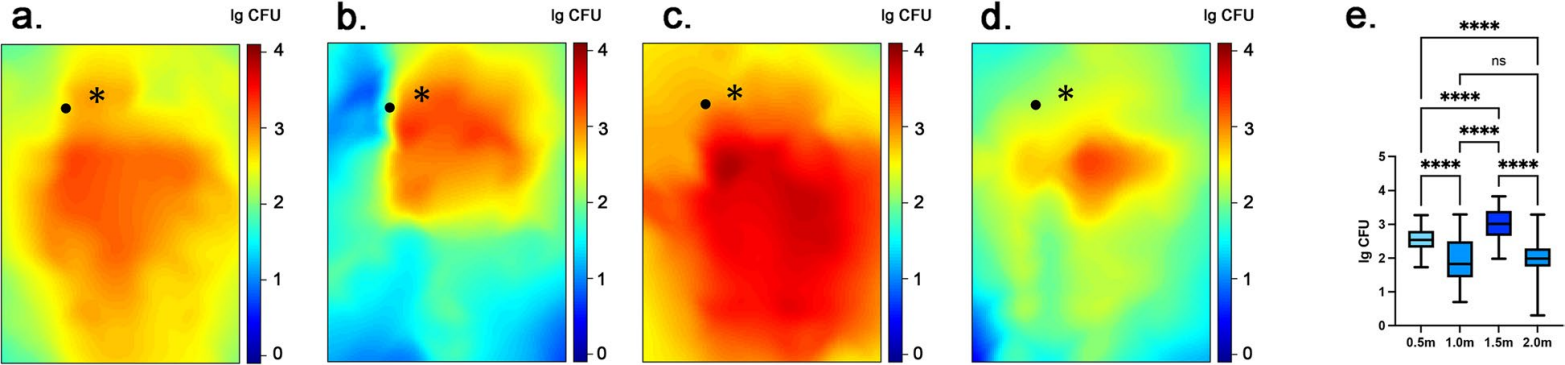
2-dimensional graphic model demonstrating the spatial distribution of biological tracer level in each height plane with a high-speed electric turbine: **a** 0.5 m. **b** 1.0 m. **c** 1.5 m. **d** 2.0 m. **e** the biological tracer level in each height plane with a high-speed electric turbine. * in each graph represents the location of the infection source, that is, the mouth of the dummy head. • in each graph represents the location of the operator

**Fig. 6:**
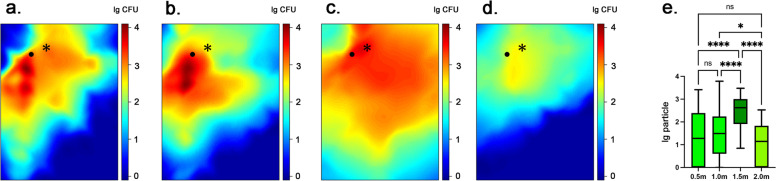
2-dimensional graphic model demonstrating the spatial distribution of biological tracer level in each height plane with an ultrasonic device: a 0.5 m. b 1.0 m. c 1.5 m. d 2.0 m. **e** the biological tracer level in each height plane with an ultrasonic device. * in each graph represents the location of the infection source, that is, the mouth of the dummy head. • in each graph represents the location of the operator

Figures 4, 5 and 6 shows the three-dimensional spatial distribution of bacterial tracers after using a high-speed air turbine, a high-speed electric handpiece, and an ultrasonic device in the dental treatment room. It’s obvious that the bacterial tracer distribution shows higher concentration after using a high-speed air turbine and a high-speed electric handpiece. Although it shows a lower tracer’s concentration with a high-speed electric handpiece. The minimum tracer concentration is in the ultrasonic device. In addition, Figs. 4 and 5 showed the distribution of the tracer in the planes of different heights with high-speed turbines. The bacterial tracer could be detected in nearly the whole space of the dental treatment room after the simulated tooth preparation. Among the 4 different heights, high-speed air turbine handpieces and high-speed electric turbine handpieces showed similar results in the spatial distribution. The 1.5 m plane exhibited the highest mean level of tracer (3.32 ± 0.43 lg CFU, 3.03 ± 0.44 lg CFU), followed by the 0.5 m plane (2.87 ± 0.35 lg CFU, 2.55 ± 0.35 lg CFU), 1.0 m plane (2.34 ± 0.59 lg CFU, 2.01 ± 0.63 lg CFU) and 2.0 m plane (2.31 ± 0.38 lg CFU, 2.00 ± 0.46 lg CFU), with air turbines and electric turbines respectively. The plane of 1.5 m demonstrated a significantly higher tracer level than that of 0.5 m, 1.0 m, and 2,0 m planes (*p* < 0.05). The plane of 0.5 m showed a significantly higher tracer level than that of 1.0 m, and 2.0 m planes (*p* < 0.05). But no significant difference was observed between the 1.0 m plane and the 2.0 m plane (Figs. 4e and 5e). With the ultrasonic device, the result was a little different. The 1.5 m plane exhibited the highest mean level of tracer (2.47 ± 0.70 lg CFU), followed by the 1.0 m plane (1.51 ± 1.07 lg CFU), 0.5 m plane (1.33 ± 1.15 lg CFU) and 2.0 m plane (1.17 ± 0.84 lg CFU) (Fig. 6). The plane of 1.5 m demonstrated a significantly higher tracer level than that of 0.5 m, 1.0 m, and 2.0 m planes (*p* < 0.05). The plane of 1.5 m showed a significantly higher tracer level than that of 2.0 m planes (*p* < 0.05). But no significant difference was observed between the 0.5 m plane and the 1.0 m or 2.0 m plane (Fig. 6e). Analyzing the spatial distribution map of the biological tracer level in each height plane, the tracer level decreases with the increase of the distance from the infection source. However, the tracer presence at the 2.0 height plane near the ceiling was low and showed a relatively regular distribution, except for the ceiling area just over the dental unit, where we found a high tracer presence. Sampling sites behind the operator showed very low tracer presence.

**Fig. 7:**
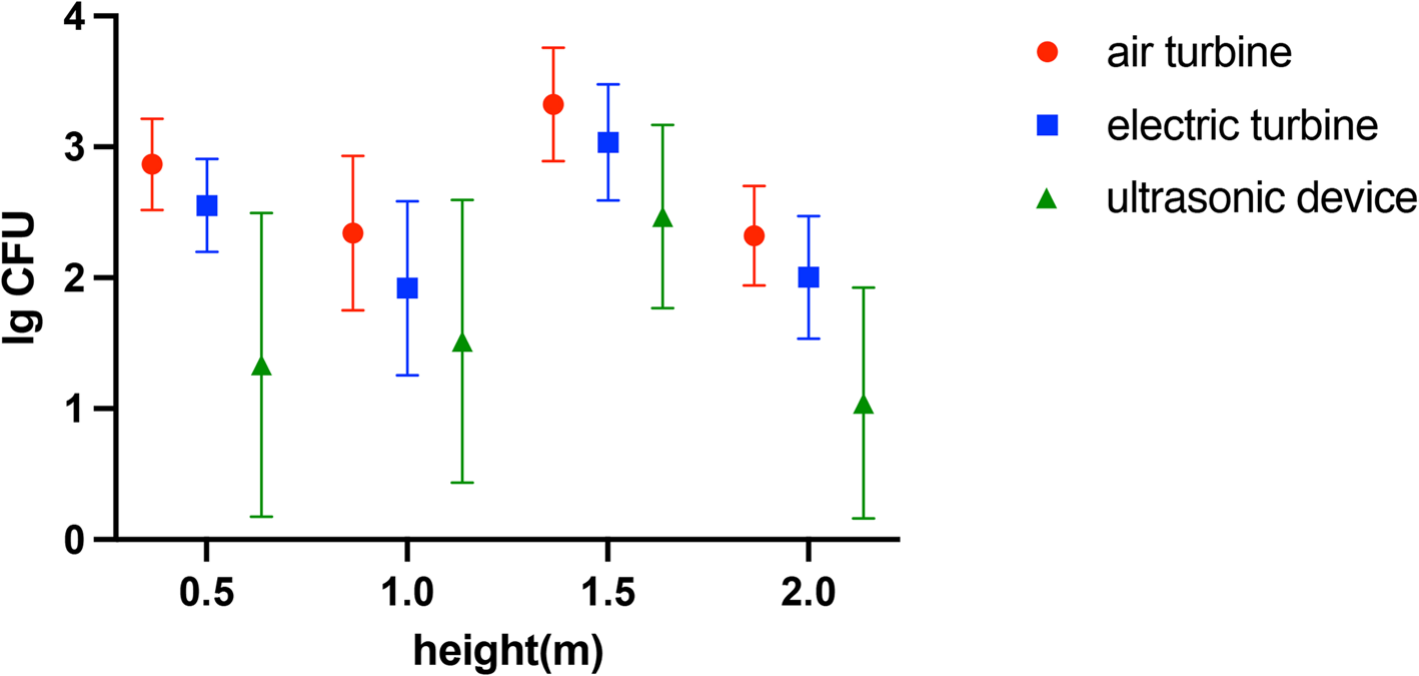
Levels of biological tracer particles (mean, ± SD, *n* = 63) in different height planes (0.5 m, 1.0 m, 1.5 m, 2.0 m) after 15-min simulated tooth preparation were compared (*p* < 0.05)

Additionally, at the four different high planes, the use of the high-speed air turbine showed the highest concentration of the biological tracer, followed by the electric turbine and ultrasonic devices (*p* < 0.05). As shown in Fig. 7, the error bar of biological tracer concentration with ultrasonic devices is bigger than in high-speed turbines due to no tracer found at the border of the operation unit. However, the tracer concentration of the operation center is higher than rotational instruments in the 0.5 m and 1.0 m heights.

### Dissipation time of microbial aerosol in the dental treatment room

#### With air a high-speed air turbine

**Fig. 8:**
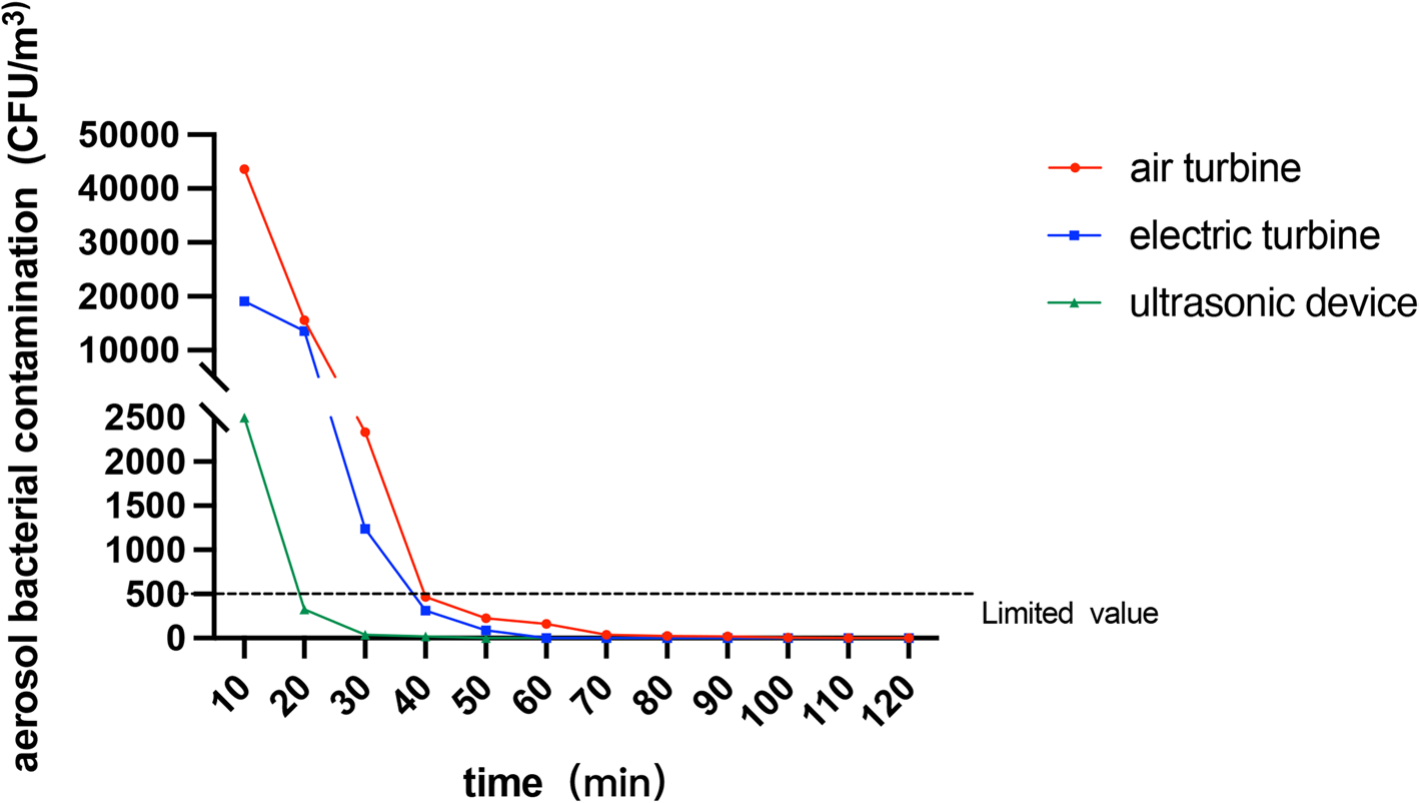
The airborne microbial contamination level (mean, *n* = 8) within 2 h after simulated tooth preparation with different instruments (*p* < 0.05)

There was a significant difference in microbial aerosol between the groups with different instruments (*p* < 0.0001). The group with air turbines showed the highest microbial aerosol contamination level peak value. As shown in Fig. 8, microbial aerosol contamination gradually decreased with the increase in dissipation time. The majority of the post dental procedure contamination was detected within the 0–10-min period and it decreased rapidly within 30 min. The contamination is reduced by 64.3% and 85.0% in 10–20-min and 20–30-min periods respectively which slowed down and tended to be flat after 30 min. According to the hygienic standard for disinfection in hospitals, the number of bacterial colonies in the air of the dental operatory should be ≤ 500 CFU/m^3^ [30]. The number of *S. mutans* colonies decreased to 500 CFU/m^3^ between 30–40 min after operation under dissipation freely.

#### With a high-speed electric handpiece

The peak value of containment concentration is lower than the air turbine. The peak value also is observed in the first 10 min. And the concentration was reduced by 29.0% in 10–20-min and 90.9% in 20–30-min periods. At 40 min after the production, the containment level was 1.6% of the original. After 30 min, the number of bacterial colonies in the air of the dental operatory is smaller than 500 CFU/m^3^. And no bacterial colonies could be found 60 min later.

#### With ultrasonic instrument

The peak value of containment concentration is much smaller than the other two rotational devices. And the biological tracers can’t be caught after 50 min. And after 10 min, the concentration of microbial meets the requirements of clinical health (Fig. 8).

### Size of microbial aerosol particles in the dental treatment room

**Fig. 9:**
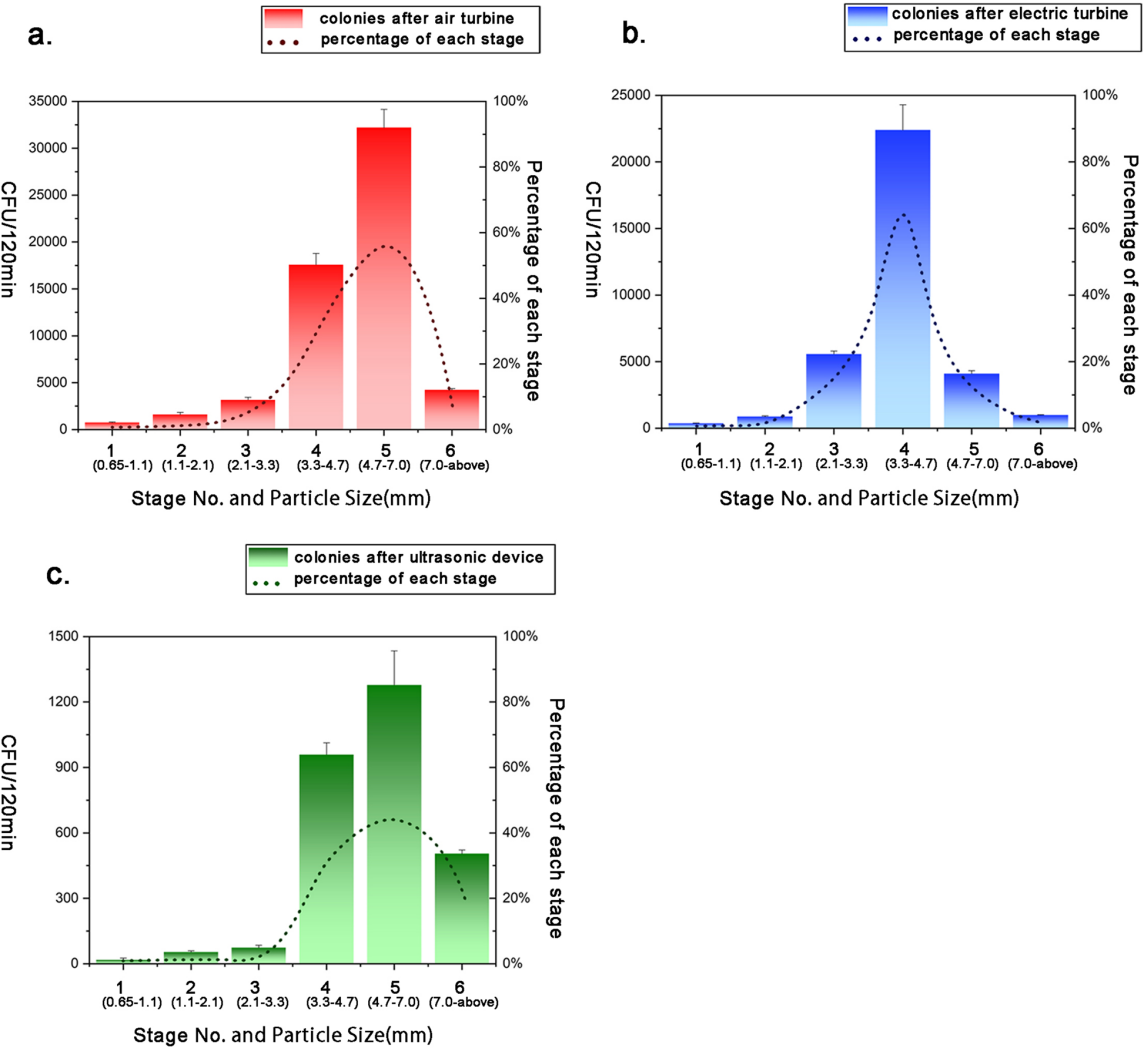
The microbial aerosol particles in six stages of Andersen microbial sampler within 2 h after simulated tooth preparation with different instruments (mean, ± SD, *n* = 3). **a** air turbine **b** electric turbine **c** ultrasonic device

With air turbines, 91% of biological tracers distribute on stage III to V, but 9% in stage I,II and VI. Most of it is on stage V(55%), followed by stage IV(30%), stage VI(7%), stage III (6%), stage II (1%), and stage I (1%) (Fig. 9a). With electric handpieces, 93% of biological tracers distribute on stage III to V, but 1% of it on stage I, 1% on stage II, and 7% on stage VI. Moreover, most tracer distributes in stage IV(56%). 1% of tracer was found in stage I which is the least (Fig. 9b). With ultrasonic devices, the most tracer is found on stage V(44%), followed by stage IV(33%), stage VI (17%), stage III (3%), stage II (2%), and stage I (1%). 94% of it is founded on stage III-IV (Fig. 9c).

## Discussions

Dental droplets and aerosols are essential potential transmission for various microorganisms [31–33]. Understanding the three-dimensional spatial distribution and dissipation time of dental airborne contamination is important for providing oral health care.

In this 3D experiment, the model measures aerosols and splatters above the plates, that can deposit on the plates. It was observed that most CFU was collected with high-speed air turbine and most CFU were collected at a height of 1.5 m. Most CFU were concentrated around the dental chair, and the least polluted areas were all behind the operator because that operator’s body will influence the transmissions of splatters, droplets, and aerosols. The size of the droplets and aerosols are mostly with 1.1 and 4.7 μm range while these by using an ultrasonic device are smaller (0.65—3.3 μm). Particles smaller than 5 μm have a higher infectious risk because they can cause lower respiratory tract infections in humans [34, 35]. In this study, all three equipment types generated small particles(< 5 μm). Since the number of bacteria per aerosol or splatter particle may differ, the exact number of bacteria cannot be established by CFU. We should realize that the infection risk in larger particles may be higher due to the larger number of bacteria.

The results of this study underestimated the propagation range and consistent time of dental airborne contamination because only bacterial contamination was determined [36]. For example, viruses are much smaller and they could have longer residence times in the atmosphere and, consequently, will be dispersed further [37, 38]. And the oral biofilm which is more complex was not considered in this study [39]. Other limitations of our investigations are related to the setting which provides space constraints to the spread of aerosols, and the maximum range of contamination spatial distribution is not obtained. Also, the door, windows, and air condition were all closed and the impact of airflow on airborne contamination distribution in clinical practice is not considered. The subject of the three-dimensional spatial distribution experiment was only a single-chair clinic with no involve multi-chair clinic involved. In this study, there are also some shortcomings because the model wasn’t equivalent to a real dental procedure. We used a mannequin rather than the clinic practice. The movement of the handpiece or the patients cannot be represented in this model. We can draw limited conclusions. Then, discrimination between small and larger droplets should have been made.

It was reported that splatter/aerosol distribution from dental procedures in an open plan clinic [40]. Similar results were shown in our study that the pollution was detected at up to 2 m. It was observed that the high-speed electric handpieces generated fewer bacteria contamination compared with high-speed air turbines in a previous study [41]. Its possible reason is that high-speed electric handpieces have a more stable speed and torque, and lower rotation speed in addition. The maximum free-running revolutions per minute (rpm) rate of the electric handpiece is generally lower than that of the air turbine (200,000 versus 400,000) [42]. Moreover, the airflow contributes significantly to the production of droplets and aerosols when using an air turbine, that spread farther. The atomization will influence the contamination level with the turbine use [13, 43].

In previous studies, it was concluded that a period of 2 h without dental treatment was sufficient to return to baseline, which prevented cumulative contamination [44, 45]. Richard Holliday and others concluded that very little additional settled aerosol microbial contamination can be detected between 30 and 60 min after the procedure [14, 40, 45]. Different sampling methods, sampling sites, culture conditions, and dental treatment room structure may affect the research results, so it is difficult to quantitatively compare the results between different studies. In this study, the baseline level is reached within 2 h after the procedure and contamination can persist in the air for up to 30 min after the procedure. Therefore a 30-min interval is recommended after a previous operation during an epidemic [22, 40]. Dental offices also should be equipped with high volume aspirations or UV disinfection lamps to shorten the time of high-risk airborne microbial contamination and reduce the risk of cross infections according to the findings of previous research [46–48]. Some studies suggest that technologies combine gravity settling, air circulation, and air filtration for particle removal [26]. Additionally, some equipment with biological sterilization also can be used in clinical but its security requires attention. Patients also need preprocedural mouth rinsing to reduce aerosol contamination [49]. Personal protective equipment (PPE) is also important for medical personnel [50, 51].

In conclusion, this study has revealed the distribution of bacterial contamination in dental offices. The bacterial droplets and aerosols tend to concentrate at the 1.5 m height. The droplets and aerosols generated by ultrasonic devices were fewer and smaller than that of high-speed turbines.

## Data Availability

All data generated or analysed during this study are included in this published article. The original datasets are available from the corresponding author on reasonable request.

## Abbreviations

DT: Dissipation time
AMS: Andersen microbial sampler
*S. mutans*: *Streptococcus mutans*

## References

[1] Volgenant CMC, de Soet JJ (2018). Cross-transmission in the dental office: does this make you Ill?. Curr Oral Health Rep.

[2] Conway DI, Culshaw S, Edwards M, Clark C, Watling C, Robertson C, Braid R, O’Keefe E, McGoldrick N, Burns J (2021). SARS-CoV-2 positivity in asymptomatic-screened dental patients. J Dent Res.

[3] Laheij AMGA, Kistler JO, Belibasakis GN, et al. Healthcare-associated viral and bacterial infections in dentistry. J Oral Microbiol. 2012;4:17659. 10.3402/jom.v4i0.17659 PMC3375115 22701774

[4] Matys J, Grzech-Leśniak K (2020). Dental aerosol as a hazard risk for dental workers. Materials.

[5] Kumar PS, Subramanian K (2020). Demystifying the mist: Sources of microbial bioload in dental aerosols. J Periodontol.

[6] Dudding T, Sheikh S, Gregson F, Haworth J, Haworth S, Main BG, Shrimpton AJ, Hamilton FW, Ireland AJ, Group A (2022). A clinical observational analysis of aerosol emissions from dental procedures. PLoS one.

[7] Peng X, Xu X, Li Y, Cheng L, Zhou X, Ren B (2020). Transmission routes of 2019-nCoV and controls in dental practice. Int J Oral Sci.

[8] Tang JW, Bahnfleth WP, Bluyssen PM, Buonanno G, Jimenez JL, Kurnitski J, Li Y, Miller S, Sekhar C, Morawska L (2021). Dismantling myths on the airborne transmission of severe acute respiratory syndrome coronavirus-2 (SARS-CoV-2). J Hosp Infect.

[9] Xu R, Cui B, Duan X, Zhang P, Zhou X, Yuan Q (2020). Saliva: potential diagnostic value and transmission of 2019-nCoV. Int J Oral Sci.

[10] Li Y, Ren B, Peng X, Hu T, Li J, Gong T, Tang B, Xu X, Zhou X (2020). Saliva is a non-negligible factor in the spread of COVID-19. Mol Oral Microbiol.

[11] Szymańska J (2007). Dental bioaerosol as an occupational hazard in a dentist’s workplace. Ann Agric Environ Med.

[12] Persoon IF, Volgenant CMC, van der Veen MH, Opdam NJM, Manton DJ, Bruers JJM. Impact of the Coronavirus on Providing Oral Health Care in the Netherlands. Int Dent J. 2022;72(4):545-51. 10.1016/j.identj.2021.09.003 PMC8452527 34706826

[13] Allison JR, Edwards DC, Bowes C, Pickering K, Dowson C, Stone SJ, Lumb J, Durham J, Jakubovics N, Holliday R (2021). The effect of high-speed dental handpiece coolant delivery and design on aerosol and droplet production. J Dent.

[14] Hallier C, Williams DW, Potts AJ, Lewis MA (2010). A pilot study of bioaerosol reduction using an air cleaning system during dental procedures. Br Dent J.

[15] Vilarinho Oliveira AMA, de Alencar RM, Santos Porto JC, Fontenele Ramos IRB, Noleto IS, Santos TC, Mobin M (2018). Analysis of fungi in aerosols dispersed by high speed pens in dental clinics from Teresina, Piaui, Brazil. Environ Monit Assess.

[16] Watanabe A, Tamaki N, Yokota K, Matsuyama M, Kokeguchi S (2018). Use of ATP bioluminescence to survey the spread of aerosol and splatter during dental treatments. J Hosp Infect.

[17] Melzow F, Mertens S, Todorov H, Groneberg DA, Paris S, Gerber A (2022). Aerosol exposure of staff during dental treatments: a model study. BMC Oral Health.

[18] Polednik B (2014). Aerosol and bioaerosol particles in a dental office. Environ Res.

[19] Veena HR, Mahantesha S, Joseph PA, Patil SR, Patil SH (2015). Dissemination of aerosol and splatter during ultrasonic scaling: a pilot study. J Infect Public Health.

[20] Yang M, Chaghtai A, Melendez M, Hasson H, Whitaker E, Badi M, Sperrazza L, Godel J, Yesilsoy C, Tellez M (2021). Mitigating saliva aerosol contamination in a dental school clinic. BMC Oral Health.

[21] Ionescu AC, Cagetti MG, Ferracane JL, Garcia-Godoy F, Brambilla E (2020). Topographic aspects of airborne contamination caused by the use of dental handpieces in the operative environment. J Am Dent Assoc.

[22] Allison JR, Currie CC, Edwards DC, Bowes C, Coulter J, Pickering K, Kozhevnikova E, Durham J, Nile CJ, Jakubovics N (2021). Evaluating aerosol and splatter following dental procedures: Addressing new challenges for oral health care and rehabilitation. J Oral Rehabil.

[23] Zemouri C, Volgenant CMC, Buijs MJ, Crielaard W, Rosema NAM, Brandt BW, Laheij A, De Soet JJ (2020). Dental aerosols: microbial composition and spatial distribution. J Oral Microbiol.

[24] Shahdad S, Patel T, Hindocha A, Cagney N, Mueller J-D, Seoudi N, Morgan C, Din A. The efficacy of an extraoral scavenging device on reduction of splatter contamination during dental aerosol generating procedures: an exploratory study. Br Dent J. 2020;1-10. 10.1038/s41415-020-2112-7 PMC7484927 32918060

[25] Takanabe Y, Maruoka Y, Kondo J, Yagi S, Chikazu D, Okamoto R, Saitoh M (2021). Dispersion of aerosols generated during dental therapy. Int J Environ Res Public Health.

[26] Razavi M, Butt ZA, Chen H, Tan Z (2021). In situ measurement of airborne particle concentration in a real dental office: implications for disease transmission. Int J Environ Res Public Health.

[27] Montebugnoli L, Dolci G (2000). Effectiveness of two devices designed to prevent fluid retraction in a high-speed handpiece. J Prosthet Dent.

[28] Andersen AA, Andersen MR (1962). A monitor for airborne bacteria. Appl Microbiol.

[29] Andersen AA (1958). New sampler for the collection, sizing, and enumeration of viable airborne particles. J Bacteriol.

[30] China. Beijing. SAotPsRo: hygienic standard for disinfection in hospitals In. vol. GB 15982–1995. Beijing: Standards Press of China; 1995.

[31] Harrel SK, Molinari J (2004). Aerosols and splatter in dentistry: a brief review of the literature and infection control implications. J Am Dent Assoc.

[32] Cristina ML, Spagnolo AM, Sartini M, Dallera M, Ottria G, Lombardi R, Perdelli F (2008). Evaluation of the risk of infection through exposure to aerosols and spatters in dentistry. Am J Infect Control.

[33] Jones RM, Brosseau LM (2015). Aerosol transmission of infectious disease. J Occup Environ Med.

[34] Considerations for the provision of essential oral health services in the context of COVID-19. https://www.who.int/publications/i/item/who-2019-nCoV-oral-health-2020.1.

[35] Gralton J, Tovey E, McLaws M-L, Rawlinson WD (2011). The role of particle size in aerosolised pathogen transmission: a review. J Infect.

[36] Chen X, Liao B, Cheng L, Peng X, Xu X, Li Y, Hu T, Li J, Zhou X, Ren B (2020). The microbial coinfection in COVID-19. Appl Microbiol Biotechnol.

[37] Neumann G, Kawaoka Y (2015). Transmission of influenza A viruses. Virology.

[38] Reche I, D’Orta G, Mladenov N, Winget DM, Suttle CA (2018). Deposition rates of viruses and bacteria above the atmospheric boundary layer. ISME J.

[39] Lin Y, Zhou X, Li Y (2022). Strategies for Streptococcus mutans biofilm dispersal through extracellular polymeric substances disruption. Mol Oral Microbiol.

[40] Holliday R, Allison JR, Currie CC, Edwards DC, Bowes C, Pickering K, Reay S, Durham J, Lumb J, Rostami N (2021). Evaluating contaminated dental aerosol and splatter in an open plan clinic environment: Implications for the COVID-19 pandemic. J Dent.

[41] Hall DL (2003). Methicillin-resistant Staphylococcus aureus and infection control for restorative dental treatment in nursing homes. Spec Care Dentist.

[42] Ercoli C, Rotella M, Funkenbusch PD, Russell S, Feng C (2009). In vitro comparison of the cutting efficiency and temperature production of ten different rotary cutting instruments. Part II: electric handpiece and comparison with turbine. J Prosthet Dentist.

[43] Sergis A, Wade WG, Gallagher JE, Morrell AP, Patel S, Dickinson CM, Nizarali N, Whaites E, Johnson J, Addison O (2021). Mechanisms of atomization from rotary dental instruments and its mitigation. J Dent Res.

[44] Al Maghlouth A, Al Yousef Y, Al-Bagieh NH (2007). Qualitative and quantitative analysis of microbial aerosols in selected areas within the College of Dentistry, King Saud University. Quintessence Int.

[45] Al Maghlouth A, Al Yousef Y, Al Bagieh N (2004). Qualitative and quantitative analysis of bacterial aerosols. J Contemp Dent Pract.

[46] Kumbargere Nagraj S, Eachempati P, Paisi M, Nasser M, Sivaramakrishnan G, Verbeek JH (2020). Interventions to reduce contaminated aerosols produced during dental procedures for preventing infectious diseases. Cochrane Database Syst Rev.

[47] Samaranayake LP, Fakhruddin KS, Buranawat B, Panduwawala C (2021). The efficacy of bio-aerosol reducing procedures used in dentistry: a systematic review. Acta Odontol Scand.

[48] Ravenel TD, Kessler R, Comisi JC, Kelly A, Renne WG, Teich ST (2020). Evaluation of the spatter-reduction effectiveness and aerosol containment of eight dry-field isolation techniques. Quintessence Int.

[49] Gupta G, Mitra D, Ashok KP, Gupta A, Soni S, Ahmed S, Arya A (2014). Efficacy of preprocedural mouth rinsing in reducing aerosol contamination produced by ultrasonic scaler: a pilot study. J Periodontol.

[50] Verbeek JH, Rajamaki B, Ijaz S, Sauni R, Toomey E, Blackwood B, Tikka C, Ruotsalainen JH, Kilinc Balci FS (2021). Personal protective equipment for preventing highly infectious diseases due to exposure to contaminated body fluids in healthcare staff. Emergencias.

[51] Patil S, Moafa IH, Bhandi S, Jafer MA, Khan SS, Khan S, Carroll WB, Awan KH (2020). Dental care and personal protective measures for dentists and non-dental health care workers. Dis Mon.

